# There is no age limit for methadone: a retrospective cohort study

**DOI:** 10.1186/1747-597X-6-9

**Published:** 2011-05-18

**Authors:** Kenneth M Dürsteler-MacFarland, Marc Vogel, Gerhard A Wiesbeck, Sylvie A Petitjean

**Affiliations:** 1 Psychiatric Hospital of the University of Basel, Division of Substance Use Disorders, Wilhelm Klein-Strasse 27, 4012 Basel, Switzerland; 2 Psychiatric University Hospital of Zurich, Division of Substance Use Disorders, 8002 Zurich, Switzerland

## Abstract

**Background:**

Data from the US indicates that methadone-maintained populations are aging, with an increase of patients aged 50 or older. Data from European methadone populations is sparse. This retrospective cohort study sought to evaluate the age trends and related developments in the methadone population of Basel-City, Switzerland.

**Methods:**

The study included methadone patients between April 1, 1995 and March 31, 2003. Anonymized data was taken from the methadone register of Basel-City. For analysis of age distributions, patient samples were split into four age categories from '20-29 years' to '50 years and over'. Cross-sectional comparisons were performed using patient samples of 1996 and 2003.

**Results:**

Analysis showed a significant increase in older patients between 1996 and 2003 (p < 0.001). During that period, the percentage of patients aged 50 and over rose almost tenfold, while the proportion of patients aged under 30 dropped significantly from 52.8% to 12.3%. The average methadone dose (p < 0.001) and the 1-year retention rate (p < 0.001) also increased significantly.

**Conclusions:**

Findings point to clear trends in aging of methadone patients in Basel-City which are comparable, although less pronounced, to developments among US methadone populations. Many unanswered questions on medical, psychosocial and health economic consequences remain as the needs of older patients have not yet been evaluated extensively. However, older methadone patients, just as any other patients, should be accorded treatment appropriate to their medical condition and needs. Particular attention should be paid to adequate solutions for persons in need of care.

## Background

The prevalence of opioid dependence is relatively stable in most European countries, including Switzerland, and currently stands at 0.1%-0.8% [1,2]. Opioid dependence is a serious chronic condition that comes with a multitude of health and psychosocial risks and one which requires adequate long-term treatment [3-5]. Based on decades of research, maintenance treatment with methadone or other suitable opioids, such as buprenorphine and slow-release morphine, is currently the mainstay of therapy for opioid dependence [6-8]. Opioid maintenance is effective, well-tolerated and internationally accepted; it is also one of the most widely studied treatments for any disease [5,9,10]. Given adequate dosage and regular dosing, maintenance medication can prevent withdrawal symptoms, curb craving for heroin as well as other opioids and block their rewarding effects [8,11,12]. Maintenance treatment also substantially reduces health and psychosocial risks associated with illicit opioid use [13-15]. Ideally, it would be a comprehensive treatment integrating medical care, psychosocial counselling and other services in which maintenance medication is prescribed to opioid-dependent persons without any undue restrictions [16,17].

Since its inception over 45 years ago, hundreds of thousands of opioid users worldwide have benefited from methadone maintenance [5,7]. While a new generation of opioid-dependent persons is entering treatment for the first time, at the other end of the age spectrum the population of older long-term patients continues to grow rapidly [18-21]. This is largely due to the success of methadone programs in retaining patients in treatment and helping to prolong their lives--all that in spite of often complex and comorbid cases [20,22-25]. Furthermore, Arndt et al. recently found increasing proportions of patients aged older than 55 mentioning problematic heroin use when presenting for first-time treatment of substance use in the US [26].

There are many references in geriatric literature to methadone as it relates to the management of chronic and cancer pain. However, little information is available about opioid use and maintenance treatment in the elderly as well as their specific needs [20,24,25]. In addition, virtually no research addresses developments of the age structure of methadone populations. Among the exceptions are reports from the Addiction Treatment Forum. Data from "Beth Israel Healthcare System", New York City--the largest methadone program in the US with over 6,000 patients--points to remarkable trends and developments in the years between 1975 and 2002 [27]. The proportion of patients aged 50 or over significantly increased during the 27-year period to encompass more than one third (35.3%) of patients in 2002. Nearly 6.5% were 60 or older. During the same time, the proportion of patients aged 39 and under steadily declined. Data from other methadone centers in the US show comparable figures. According to the US Department of Health and Human Services [21], almost 10% of methadone patients were over the age of 50 at the end of the last decade, and more than 33% were between the ages of 40 and 49. While this data points to clear trends in aging methadone patients in the US, there is great variability across treatment centers. In a survey among ten methadone programs from nine states, estimates of the percentage of patients aged 55 or older ranged from 2% to 60% [18]. This raises the questions of why the discrepancy exists and whether these developments are restricted to the US or whether there are similar trends in other countries with relatively long traditions of methadone maintenance and rather high prevalence rates of opioid dependence. Slowly, these issues also attract attention in Europe, as demonstrated by the creation of collaborative projects like "SDDCare" to explore needs and establish treatment guidelines for senior drug users http://www.sddcare.eu.

In Switzerland, the prescription of methadone for opioid dependence has been regulated by federal and state laws since 1975. Methadone maintenance treatment (MMT) is covered by health insurance and is provided by specialized clinics and office-based practitioners authorized to perform this treatment [28]. It is available for opioid-dependent individuals (according to ICD-10 criteria) aged 18 years or older who show opioid use on toxicology screening but are not able or willing to undergo abstinence-oriented treatment. Patients are generally free to choose their physician and treatment is covered by mandatory health insurance. Swiss health authorities substantially increased treatment programs in response to open drug scenes in several cities in the beginning of the 1990s [29]. Since then, there have been sufficient facilities for all patients willing to enter treatment. Thus, the threshold for entrance into MMT is low, with no further prerequisites other than opioid dependence. The name of every patient is reported to the local health authorities upon admission to MMT. Long-term abstinence is not an obligatory treatment goal and there are no restrictions regarding duration of treatment and dosing of methadone although currently a minimum dose of 60-80 mg/d is recommended [30]. Take-home dosages are often allowed for a maximum of one week. In March 2003, about 17,000 patients were on MMT throughout Switzerland [29]. From these, 958 were treated in Basel-City where the ratio of MMT patients to population has been roughly 0.5% since 1995. The MMT population of Basel has similar incidence trends, mean age and age of onset of heroin use as those of other cities in Switzerland [31]. We therefore assume the cohort to be representative for other Swiss cities.

This retrospective register-based cohort study sought to determine the age trends in the MMT population of Basel-City, Switzerland from April 1, 1995 to March 31, 2003, and to discuss some aspects of the potential consequences related to these developments in the future.

## Methods

### Data basis and statistics

All data was taken from the methadone register of the health authorities of Basel-City, the operation of which was discontinued in 2004. MMT data in the canton of Basel-City had been collected and evaluated since 1995. The data collection and evaluation are in accordance with the data protection law of the canton of Basel-City and were approved by the local ethics committee. As stipulated by legislation, prescribing treatment providers in Basel-City are required to submit a registration form to the health authorities each time a patient begins and ends MMT. The form collects a limited amount of information about methadone patients and their treatment but provides a population-based data source recording the duration of treatment episodes as well as the characteristics of the prescribing provider. For monitoring purposes, methadone prescribers were further invited until 2004 to provide anonymized patient and treatment data to the register every 12 months by means of a 2-page questionnaire. This structured questionnaire contained a core of unchanging questions about sex, marital status, type of housing, educational level, work situation, and self-reported substance use during the previous 30 days.

The present evaluation includes data of MMT patients from April 1, 1995 to March 31, 2003. Throughout that period, three clinics (two public, one private) and a maximum of 81 office-based practitioners conducted MMT in Basel-City. All returned questionnaires were checked for plausibility and erroneous data entries were corrected or removed if they were ambiguous. Therefore, and since not all respondents answered all questionnaire items patient numbers vary across variables. For the analysis of the developments in age structure, patient samples of the years 1996, 1997, 1998, 2000, 2001, 2002 and 2003 were split into four age categories: 20-29 years; 30-39 years; 40-49 years; and 50 years and over. Because the database for 1999 was incomplete, it was excluded. Cross-sectional comparisons were performed using patient samples of 1996 and 2003.

Statistical analysis was conducted using SPSS for Windows (release 15). Data were analyzed by chi-squared statistics, Student's t-test for unpaired samples, and analysis of variance, where appropriate. Because of multiple testing all statistical data were considered significant at p < 0.01.

## Results

**Figure 1:**
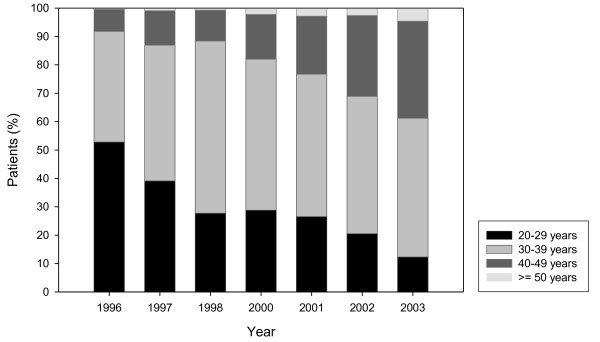
**Age trends of the MMT population in the canton of Basel-City, Switzerland, 1996 to 2003**.

The analysis of methadone treatments in Basel-City indicates a clear trend in age development of the MMT population, as depicted in Figure 1. It shows a significant increase in older patients between 1996 and 2003. During that period, percentages of persons aged 50 and over rose tenfold from 0.4% in 1996 to 4.6% in 2003 (see Table 1). During the same period, the proportion of patients aged 29 and under dropped significantly from 52.8% in 1996 to 12.3% in 2003. This trend is reflected in the average age of patients, which stood at 30.3 years in 1996 and rose to an average of 38.0 years in 2003.

**Table 1:** Socio-demographic and clinical characteristics of the methadone patient population in Basel-City, Switzerland, 1996 and 2003

Variables	Sample 1996 (n = 1,195)	Sample 2003 (n = 958)	Statistical data
Age in years, mean (SD)	30.3 (6.3)	38.0 (7.1)	T[1602,7] = 24.39; p < 0.001
Age category			Chi^2 ^[3] = 419.32; p < 0.001
20-29 yrs	52.8	12.3	
30-39 yrs	39.0	48.9	
40-49 yrs	7.8	34.2	
> 50 yrs	0.4	4.6	
Men	67.7	68.3	
Dose in mg, mean (SD)	70.4 (39.9)	80.9 (59.4)	T[1213] = 3.71; p < 0.001
Yrs in current treatment, mean (SD)	3.2 (3.2)	8.6 (4.3)	T[1296,3] = 29.12; p < 0.001
1-year retention rate	65.4	86.9	Chi^2 ^[1] = 111.13; p < 0.001
Educational level			Chi^2 ^[2] = 20.70; p < 0.001
Regular school	56.5	45.4	
Apprenticeship	40.2	51.1	
Higher education	3.3	3.5	
Employment status/source of income			Chi^2 ^[2] = 104.55; p < 0.001
Employed (unemployment ins. incl.)	48.9	24.1	
Social welfare	24.0	33.9	
Disability pension	27.1	42.0	
Heroin use in the past month	74.5	47.1	Chi^2 ^[1] = 95.37; p < 0.001
Cocaine use in the past month	36.5	49.6	Chi^2 ^[1] = 13.83; p < 0.001

Values are percentages unless otherwise indicated.

As shown in Table 1 the comparison of the patient samples of 1996 and 2003 reveals further trends as well as certain constants. The ratio of male to female patients remained virtually unchanged, whereas the proportion of employed patients working in jobs or receiving unemployment insurance payments dropped significantly from 48.9% to 24.1%. In the same period, the percentage of patients receiving a disability pension increased significantly, but the educational level also rose, overall, with more patients completing an apprenticeship or a higher education qualification. The 1-year retention rate in MMT improved greatly from 65.4% to 86.9% during this period. Similarly, data from 2003 shows an average methadone dose of 80.9 mg/d while the average methadone dose in 1996 was significantly lower, lying in the middle of the minimum effective dose range (60-80 mg). Of the 958 patients registered on March, 31, 2003, 458 persons had been on MMT continuously since 1995 or before. This amounts to almost half the 2003 sample (47.8%) while the overall number of MMT patients decreased by 19.8% over the observation period.

The data indicates that as this population ages, there is still a high rate of illicit substance use, with many patients using heroin and/or cocaine during the previous month (see Table 1). In 1996, 74.5% of the population reported using heroin during the preceding month while in 2003, 47.1% reported past-month heroin use. In contrast, the prevalence of self-reported cocaine use in the past month rose significantly from 36.5% in 1996 to 49.6% in 2003. Data from 1996 and 2003 show that patients aged 40 and older tended to report previous-month heroin use less often than younger patients (1996: 64.2 vs 75.4%, Chi2[1] = 4.08, p = 0.043; 2003: 42.2 vs 50.4%, Chi2[1] = 3.87, p = 0.049) while this was not the case for past-month cocaine use which did not differ according to age.

## Discussion

To our knowledge, this is the first register-based study addressing age trends among a MMT population in Europe. The results show a significant increase in both mean age and proportion aged 40 and older in MMT patients of Basel-City between 1996 and 2003. This trend is comparable to findings obtained in the US [18,21,27], although there have not been many of those and the present trends are less pronounced. The increase in age-coupled with a relatively stable prevalence of opioid dependence-corresponds well with the declining incidence of heroin use and the higher MMT retention rates in Switzerland [1,32]. The 1-year retention rate of 86.9% in 2003 is certainly favorable [33-35]. It reached a level of 65.4% as early as 1996, which compares well with national and international levels at that time [36-39]. Taking into account other factors (e.g. improved clinical experiences, more adequate treatment of comorbid disorders) this increase could be due to a less restrictive MMT practice and the prescription of more effective methadone doses [33]. This change in treatment practice, may have contributed to the observed increase in patients aged 40 or older that requires particular attention. Furthermore, the observed age trends may be due to the effectiveness of MMT in decreasing mortality of drug users as compared to those not in treatment.

The findings also point to another relevant trend among MMT patients. As compared to 1996, there were considerably more patients receiving a disability pension or social welfare benefits in 2003, which also may be a consequence of this population's aging and its problems related to prolonged substance use and associated lifestyle, such as comorbidity, malnutrition, loss of work, unsanitary living conditions, violence, and trauma [40-42].

Among the MMT population of Basel-City, past-month heroin use decreased significantly through time, from 74.5% to 47.1%. This could be the result of higher methadone doses and longer stays in treatment [13,28,33]. However, past-month cocaine use in this population increased from 36.5% to 49.6%. This finding is comparable to that of a study of clients of Swiss needle-sharing facilities from 1993-2006 [43]. Among other reasons, this shift from heroin to cocaine use may be the result of the decreasing price of cocaine [44] or the declining purity of street heroin which may lose attractiveness in the light of agonist properties of methadone received in treatment. New patterns of consumption have accompanied the shift, with heroin, cocaine and sometimes rapid-onset benzodiazepines used together [43]. The sustained increase in cocaine use among MMT patients contrasts with the relatively constant prevalence in the general population [45] and underlines the increased vulnerability for multiple drug use in this group.

### Treating aging methadone patients

This study confirms trends outlined in previous reports from the US showing that MMT populations are getting older with many patients aged 50 and older [18,21]. As positive as this trend is, the aging of the methadone population throws up a host of questions. Aging is a process that brings about changes that can have a profound impact on a person's health and well-being. The same applies to methadone patients, and often even more so, since many of them have aged prematurely as a result of a history of long-standing substance use [46], and they often suffer from chronic diseases [20,23,25,47,48]. Health problems resulting from prolonged substance use can accelerate the decline in health some older persons already experience. Additionally, psychosocial problems, such as diminished relationship webs as well as reduced socioeconomic resources and security, often follow. Elderly methadone patients may experience marginalization in the peer group of substance users and thus suffer from multiple stigmatization due to drug use and age [49,50]. Many reintegrative approaches in treatment of substance users are based on work or other occupation and may not be suited for this patient group. On the other hand, elderly substance users in general have been shown to profit from psychotherapy and may do so even more when this is tailored to their needs [51,52]. Depressive disorders may also be overrepresented among older MMT patients [20,23,25]. However, there has been almost no research addressing the specific problems and needs of older methadone patients. Consequently, many questions about the adequate medical care of these patients and its cost-effectiveness remain unanswered. Health care professionals, however, must address the practical treatment problems of older MMT patients, such as chronic disease, pain and disability.

From a medical perspective, older MMT patients should be accorded treatment appropriate to their age, just like any other patients-meaning methadone should be considered as a medication for the well-being of these patients without reservation. The underlying therapeutic approach must be guided by professionalism and respect, and should be tailored to the individual patient's needs. It is also worth taking into account that many patients have made negative experiences with the medical system during their substance use history and have been subject to stigmatization [49]. MMT providers must develop new approaches as they face a growing population of older patients who tend to have long treatment histories, sometimes 30 years or more [3]. Many of them are interested in their health and are amongst the most stable patients [18,24,53]. However, state regulations, existing rules and prejudices surrounding methadone may affect them and their treatment [4,54]. In Switzerland as in most other countries, take-home methadone is subject to regulations which limit take-home doses to a maximum of a couple of days, irrespective of duration and course of treatment. Even though it would be desirable to allow healthy older patients in stable treatment fewer visits of the treatment provider, take-home doses are generally limited to a maximum of one week in Basel.

On the other hand, older patients can also pose a unique set of clinical challenges related to the medical issues of aging, such as arthritis, hypertension, liver disease, obstructive pulmonary disease, osteoporosis, diabetes, and reduced mobility [20,22,23,25,55]. Thus, the cooperation between MMT providers and institutions of primary as well as secondary care is of growing importance. And, there can be complications with elderly patients when they are provided with extended take-home methadone doses. Some persons may have difficulties handling larger supplies of methadone due to neurocognitive impairment associated with aging or prolonged substance use [56,57], or because they have to take various other medications. In such cases, it would be important for MMT providers to work proactively or to turn to external help systems (e.g. family, relatives, home care services), which can offer assistance with medications and activities of daily living. Alternatively, methadone and other medications could be received from local primary care physicians or pharmacies under the direction of specialized clinics, if necessary.

As of yet, there are no proven data on age-related alterations in methadone metabolism. Methadone dose adjustments are thus not automatically necessary due to aging. However, since renal function may decline in the elderly, the dose needs to be monitored closely and adjusted if necessary. As with younger patients, dose adjustments may be required for patients taking other medications known to interact with methadone (e.g. antiviral medications, SSRIs, antiepileptics) or for patients with severe liver or kidney disease [58-60]. There is some evidence from animal experiments and treatment of chronic pain patients that opioid tolerance may develop more slowly with age [61,62]. In any case, care should be taken when increasing doses at the beginning of treatment or after omission of provision days. Due to cumulative effects, caution is needed when respiratory-depressant medications such as benzodiazepines are prescribed. The same holds true for patients with excessive alcohol intake or use of respiratory-depressant drugs [63,64]. Generally, physicians should aim for prescription of straightforward pharmaceutic combinations and dosing regimens. Clinical experience further shows that women going through menopause may request methadone dose increases. Medical staff as well as patients should be aware that uncomfortable perimenopausal symptoms, such as hot flashes, outbreaks of sweat and fatigue are often identical to opioid withdrawal symptoms [65-67]. Methadone dose increases may not always be indicated in these cases.

Standardized assessments of neurocognitive, psychosocial and medical functioning at regular, for example yearly, intervals may prove helpful in determining the appropriate level of autonomy and support in the treatment setting for the individual patient.

### Future challenges surrounding the care of methadone patients

As time passes, more methadone patients are going to require skilled nursing care. However, many health care services and nursing homes are not equipped to, and some are not prepared to, care for these patients. The latter is often based on prejudices that carry over even to patients who have long stopped using illicit drugs. The observed increase in cocaine use, however, with a substantial proportion of elderly patients using the drug may further contribute to this problematic issue. There is also still the ill-informed view that older persons who have lived a long time without taking illicit opioids should no longer need methadone. Contrary to the "maturing-out" theory [68], which suggests that opioid-dependent persons grow out of their substance use disorder as they get older, many patients still require methadone for their well-being as they age [3,69]. Some older patients may even require higher methadone doses for reasons of comedication [70]. Others may be resistant to the lowering of their long-standing dosage due to the fear of withdrawal, even if an adjustment would be indicated [22,58]. This means methadone providers are required to raise awareness for this issue and propose ways of closer cooperation with institutions and care homes. On the other hand, it is important that home care providers and skilled nursing facilities be aware of the issues involved and plan accordingly.

Our study has several limitations. The analysis did not include all patients registered for MMT in Basel-City. However, the annual rate of returned questionnaires was high (>83%) which suggests that the developments outlined above closely resemble actual trends. The study further used a retrospective approach and only considered a limited amount of information about the MMT patients of Basel-City and their treatment. It provides, however, valuable information on certain developments concerning this population and on changes in treatment practice. It also helps to optimize MMT services and to support policy makers and practitioners in decision-making and treatment planning. Finally, some data relied on an instrument which depended upon information treatment providers had, and not all providers filled in all questionnaire items.

## Conclusions

Despite these limitations, two conclusions can be drawn from the present findings. First, MMT populations are getting older, not only in the US but also in Switzerland and most likely in many other European countries. Second, MMT practice has changed in the last decades, which is corroborated by other studies [71,72] and is most likely owed to research. However, as positive as these trends are, they will pose challenges for MMT providers and the entire health care system in the near term. We would welcome increased efforts from researchers and practitioners to deal with this issue. It is time for us to develop resources and to use them to meet the special needs of this patient population, and to train staff accordingly. Increased knowledge about these persons' needs, and the special issues involved in their care, will help decrease prejudice against them, improve the quality of care, and, indeed their quality of life.

## Competing interests

The authors declare that they have no competing interests.

## Authors' contributions

KDM, GW and SP designed the study. SP was responsible for all data collection and data management. KDM and SP conducted the statistical analyses for the manuscript. KDM and MV were involved in data interpretation and drafted the manuscript. All authors contributed to and have approved the final manuscript.
